# Fibroadipogenic progenitors: a potential target for preventing breast muscle myopathies in broilers

**DOI:** 10.3389/fphys.2024.1458151

**Published:** 2024-08-13

**Authors:** Usuk Jung, Minjeong Kim, Brynn H. Voy

**Affiliations:** Department of Animal Science, University of Tennessee, Knoxville, TN, United States

**Keywords:** broiler, fibroadipogenic progenitors, myopathy, pectoralis major, white stripping, wooden breast

## Abstract

Genetic selection for high growth rate, breast muscle yield, and feed efficiency in modern broilers has been a double-edged sword. While it has resulted in marked benefits in production, it has also introduced widespread incidence of breast muscle myopathies. Broiler myopathies are phenotypically characterized by myodegeneration and fibrofatty infiltration, which compromise meat quality. These lesions resemble those of various myopathies found in humans, such as Duchenne muscular dystrophy, Limb-girdle muscular dystrophy, and sarcopenia. Fibroadipogenic progenitors (FAPs) are interstitial muscle-resident mesenchymal stem cells that are named because of their ability to differentiate into both fibroblasts and adipocytes. This cell population has clearly been established to play a role in the development and progression of myopathies in mice and humans. Gene expression studies of wooden breast and other related disorders have implicated FAPs in broilers, but to our knowledge this cell population have not been characterized in chickens. In this review, we summarize the evidence that FAPs may be a novel, new target for interventions that reduce the incidence and development of chicken breast muscle myopathies.

## Introduction

Lesions in broiler breast muscle myopathies, such as wooden breast and white striping, are characterized by infiltration of fibrous and fatty tissue in place of healthy muscle fibers. These are thought to arise from localized fiber damage that results from physiological consequences of extremely rapid muscle growth, and disruption of the normal, highly synchronized process of muscle repair. For very different reasons, fibroadipogenic lesions develop in a range of diseases and disorders in humans, where they compromise muscle strength and mobility. A novel cell type, fibroadipogenic progenitors (FAPs) has been shown to be the source of such lesions in humans, opening up new therapeutic targets to prevent this pathology. Here we review evidence that this same cell type plays a comparable role in development of broiler breast muscle myopathies.

## Myopathies are prevalent in the breast muscle of broiler chickens

Modern broilers require less feed and a shorter amount of time to reach heavier market weight than their 1970s counterparts (National Chicken Council, 2022). However, improvements have resulted in prevalence of myopathies, primarily in the pectoralis major muscle. Different types of myopathies have been described and named based on various features of lesions, including wooden breast, white striping, deep pectoral myopathy, and spaghetti meat (Petracci et al., 2019). Each alters the biochemical and morphological properties of skeletal muscle and compromises meat quality in ways that impose significant losses to the broiler industry. Lesions negatively affect the appearance and texture of broiler meat and discourage consumer acceptance, and lesioned filets are downgraded or discarded (Kuttappan et al., 2016). The incidence of lesions in broilers is widespread, with estimates of incidence in commercial flocks are as high as 90% (Tijare et al., 2016). Lesions are not restricted to a specific broiler line and occur across the globe. The US broiler industry alone has been estimated to lose ∼ $1B due to these myopathies (Barbut, 2019).

**FIGURE 1:**
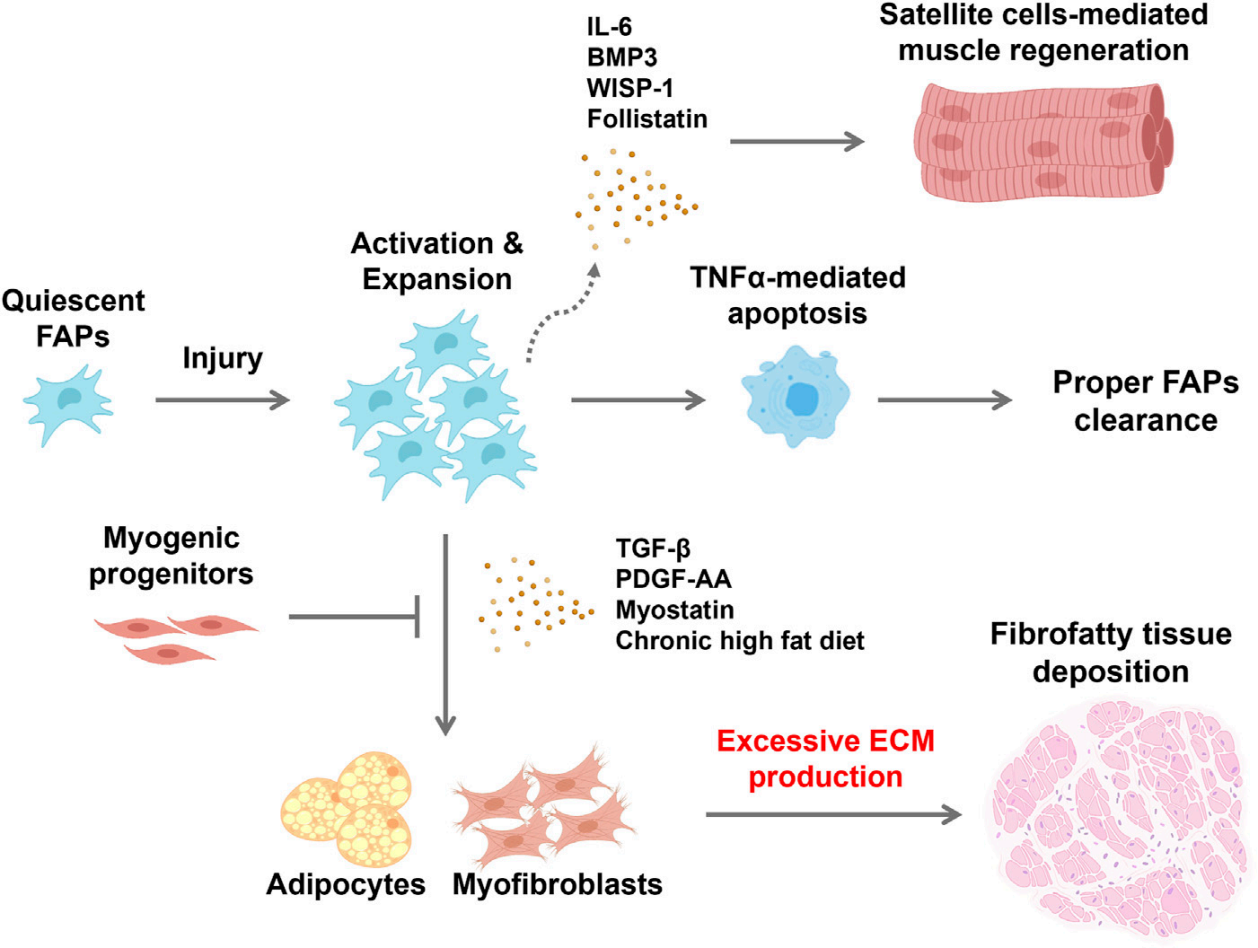
Schematic showing fibrofatty tissue infiltration in response to changes in FAP behavior within the environment of an injured skeletal muscle FAPs are normally quiescent but are activated in response to muscle injury. Proliferating FAPs release both extracellular matrix components and promyogenic cytokines to support satellite cell-mediated skeletal muscle regeneration. Satellite cells produce mediators that maintain FAPs in their undifferentiated state while repair proceeds. Immune cells that infiltrate the site of injury produce TNFα that induces apoptotic clearance of FAPs, restoring their numbers to the baseline state. However, cues within the microenvironment of dystrophic muscle can lead to persistence of FAPs and subsequent differentiation to fibroblasts and adipocytes, and fibrofatty tissue accumulation within muscle tissue. Consequently, satellite cell-mediated muscle regeneration is hampered and disrupted. Created with BioRender.com.

Wooden breast and white striping are the two most prevalent forms of myopathies in common commercial lines of broilers (Figure 1). Wooden breast lesions result when areas of the breast muscle (pectoralis major) become hardened and thickened, with hemorrhagic areas on the surface of the muscle. White striping describes white fibrotic, lipid-rich striations on the surface of the pectoralis major. These two types of lesions are interrelated and are often found together in the broiler pectoralis major muscle (Sihvo et al., 2014; Aguirre et al., 2020), and share many histological features (Velleman and Clark, 2015). Histopathologically, both types of lesions in birds at slaughter age (∼42 days) are characterized by signs of muscle fiber damage, inflammation, and fibrosis. Lesions include ectopic deposition of adipose tissue, immune cell infiltration, myofiber necrosis, degenerative fibers, accumulation of interstitial connective tissue and collagen, and severe fibrosis (Papah et al., 2017; Velleman, 2019). Numerous transcriptomic studies have described widespread differences in gene expression between muscles of affected and non-affected broilers (Zambonelli et al., 2016; Pampouille et al., 2019; Bordini et al., 2022). As would be expected from the visible features of lesions, these studies have consistently shown upregulation of genes related to adipogenesis, lipid accumulation, inflammation, and fibrosis in muscle from affected birds (Papah et al., 2017; Lake et al., 2019).

## Consequences of rapid growth that may damage muscle

Myopathies in broilers are a consequence of intense selection for extremely rapid growth of the *p. major* muscle. The incidence and severity of lesions is diminished by slowing growth through management practices, or by introducing slow-growing genetics into commercial broiler lines. Rapid growth of skeletal muscle is a desired trait in meat animals, and in and of itself does not result in myopathies or increased adipose deposition in muscle. For example, myostatin mutations that cause double muscling in cattle are associated with reduced intramuscular fat (Hanset et al., 1982). In broilers, however, rapid growth specifically of the *p. major* muscle is thought to damage myofibers and compromises muscle repair, resulting in fibrofatty infiltration that characterizes wooden breast and other myopathies (Mann et al., 2011). Several causes of myofiber damage and development of myopathies have been proposed, such as deficiency of antioxidants, alterations in lipid and glucose metabolism, and tissue hypoxia (Bordini et al., 2022; Malila et al., 2022; Velleman, 2022). A prevailing hypothesis is that fiber damage results from a very rapid rate of muscle growth that outstrips the ability of the vascular system to deliver oxygen and nutrients and remove metabolic wastes (Kuttappan et al., 2013). This inadequate perfusion is thought to result in localized damage to myofibers, initiating a cascade of failed regeneration. A relative deficit in capillary density relative to myofiber area in pectoralis major has been described as an early feature of wooden breast, suggesting that reduced blood supply to myofibers precedes the development of focal lesions at 2 weeks of age (Sihvo et al., 2018). Reduced vascularization compared to the needs of the muscle compromises the ability of the circulatory system to effectively deliver oxygen and nutrients and remove metabolic wastes such as lactic acid that can damage myofibers (Velleman, 2015). These structural changes are thought to further cause localized hypoxia within myofibers that induces inflammation and oxidative stress production, which eventually affect skeletal muscle homeostasis (Mutryn et al., 2015; Jung et al., 2024)

## Early changes during development of myopathies in broiler breast muscle

One of the major challenges in identifying the factors that cause wooden breast and other myopathies is that incidence is not detected until slaughter and processing. Papah et al. (2017) conducted a novel prospective study in which they used a needle biopsy technique to sample breast muscle tissue over time in a large number of birds from 1 week of age to market age. They identified which birds went on to develop lesions at slaughter and then compared changes in histology and gene expression over time between affected and unaffected birds. They found that localized lipogranulomas and lipid infiltration between myofibers were detectable as early as 1 week of age in birds that eventually developed wooden breast lesions. By 2–3 weeks of age affected birds showed signs of single myofiber degeneration and inflammation, and increased expression of genes involved in lipid metabolism and adipogenesis (Papah et al., 2017). Similar initial changes have been reported for development of white striping (Griffin et al., 2018). Collectively, these findings implicate lipid infiltration in the early etiology of breast muscle myopathies in broilers.

## Similarities between myopathies in human and broiler

In the context of poultry production, skeletal muscle myopathies are primarily restricted to the pectoralis major muscle of broilers due to its uniquely disproportionate rate of growth. In contrast, fibrous and fatty lesions within muscle are shared by a wide range of diseases and disorders that compromise muscle function in humans (Theret et al., 2021). Infiltration of adipose and fibrotic tissue in place of healthy muscle occurs in heritable muscular dystrophies such as Duchenne Muscular Dystrophy (DMD). In this disease, the most common muscular dystrophy, loss of the protein dystrophin compromises cytoskeletal integrity and results in progressive muscle wasting (Mendell and Lloyd-Puryear, 2013). Adipose and fibrous deposition with muscle also occurs secondarily to a range of physiological conditions, including aging, obesity, and Type 2 diabetes (Brack and Rando, 2007; Lukjanenko et al., 2019; Uezumi et al., 2021). Repeated injury and repair to the muscle itself can also result in lesions that histologically resemble those of broiler breast. For example, rotator cuff tears often occur incrementally and result in fibrofatty deposits that influence the functional outcome of surgical repair (Meyer et al., 2004). Clinically, these pathological changes to the composition of skeletal muscle are a major concern because they compromise muscle strength and mobility.

## Fibroadipogenic progenitors

Satellite cells are committed to a myogenic fate, but as a mesenchymal cell they have been shown to have some capacity for differentiation to adipocytes and other lineages (Yin et al., 2013). Accordingly, this population was initially thought to be responsible for the fibrofatty infiltration of myopathic lesions as a result of inappropriate differentiation into adipocytes and myofibroblasts. However, this role is now attributed to FAPs, a non-myogenic cell population that was identified by two independent research groups in 2010. FAPs were originally isolated from single cell isolates of healthy skeletal muscle based on expression of mesenchymal surface markers and their capacity to spontaneously differentiate into adipocytes and fibroblasts *in vitro* (Joe et al., 2010; Uezumi et al., 2010). Each group demonstrated that FAPs transiently proliferate but remain undifferentiated in muscle undergoing healthy regeneration, but they undergo adipogenic differentiation in a degenerative muscle environment. FAPs arises from a lineage that is distinct from that of satellite cells, does not express Pax7, and is not capable of myogenic differentiation. FAPs are characterized by surface expression of platelet-derived growth factor receptor alpha (PDGFRα), a membrane-bound tyrosine kinase receptor (Contreras et al., 2021) that regulates their proliferation. They reside in the interstitial space and are relatively abundant in skeletal muscle, as PDGFRα^+^ cells account for 5%–15% of the total nuclei and 20%–30% of the interstitial mononuclear cells in adult muscles (Contreras et al., 2021). Lineage tracing studies *in vivo* have confirmed that FAPs are the primary, if not the only source of adipocytes that develop in damaged muscle and yield fat infiltrates that characterize myopathic lesions (Kopinke et al., 2017).

## FAPs are required for healthy muscle repair

FAPs were initially discovered based on their role in muscle pathology. However, they are now understood to also play a critical role in muscle repair through their interactions with muscle stem cells and immune cells. Repair of muscle damage requires a series of coordinated steps and interactions between multiple cell types. Upon damage, both satellite cells and FAPs are promptly activated and enter the cell cycle, with expansion of FAPs preceding that of satellite cells. Rapid proliferation of FAPs is mediated in part through paracrine effects of PDGF that is released by invading macrophages (Contreras et al., 2019). Proliferating FAPs secrete a cocktail of cytokines and promyogenic factors, including IL-6, IL-10, IL-33, BMP3, WISP-1, and follistatin, that promote satellite cell differentiation and myofiber maturation (Joe et al., 2010; Fiore et al., 2016; Biferali et al., 2019; Wosczyna et al., 2019). In addition, FAPs are the primary source of extracellular matrix components, including collagen, metalloproteinases, fibronectin, laminin, and proteoglycans that are necessary for new muscle fiber formation (Arnold et al., 2007; Wynn, 2008). In turn, myofibers provide paracrine signals that inhibit adipogenesis and maintain FAPs in their undifferentiated state (Murphy et al., 2011; Fiore et al., 2016). The rapidly expanded population of FAPs is transient. As muscle regeneration proceeds, FAPs are induced to undergo apoptosis through the actions of TNF-α secreted from pro-inflammatory M1 macrophages recruited to the site of injury, restoring the population back to its original level (Natarajan et al., 2010; Lemos et al., 2015). The critical role of FAPs in this process, and ultimately in maintenance of healthy muscle tissue, has been demonstrated by their genetic deletion. Loss of FAPs over time leads to muscle atrophy, with decreased muscle mass, reduced fiber size, and diminished satellite cell pool (Petrilli et al., 2020). Therefore, the supportive role of FAPs in muscle homeostasis is key for maintaining the satellite cell pool and the long-term capacity for growth and repair.

## Aberrant differentiation of FAPs in dystrophic muscle

Aberrant differentiation of FAPs to fibroblasts or adipocytes results from cues that arise within the muscle environment and disrupt the tightly orchestrated balance of signals that govern regeneration, yielding the fibrofatty infiltrations that are characteristic of myopathies (Uezumi et al., 2010; Uezumi et al., 2011; Mozzetta et al., 2013; Lemos et al., 2015; Kopinke et al., 2017; Buras et al., 2019; Hogarth et al., 2022). This was initially demonstrated by Uezumi et al. (2010) upon the identification of FAPs. Activated FAPs were shown to undergo adipogenic differentiation when residing in a dystrophic muscle environment, but to remain undifferentiated when transplanted into muscle undergoing healthy repair. The specific signals that drive fibrogenesis and adipogenesis in muscle *in vivo* are still poorly understood but are of key therapeutic interest. Several secreted factors, including TGF-β, PDGF-AA, myostatin, and wnt3A, have been shown to induce fibroblastic differentiation of FAPs. TGF-β is known as a hallmark of fibrosis and is a secreted protein of macrophages, FAPs, and regenerating myofibers in skeletal muscle. TGF-β promotes fibrogenic differentiation of FAPs (Klingberg et al., 2014; Contreras et al., 2019; Lodyga and Hinz, 2020) by activating the canonical Wnt/β-catenin signaling pathway, which can also suppress adipogenesis upon nuclear translocation of β-catenin (Akhmetshina et al., 2012; Reggio et al., 2020). Signaling through the Notch, retinoic acid receptor, and Hedgehog pathways also mediates FAPs fate decisions. Notch signaling is thought to play a pivotal role in preventing adipogenesis of FAPs. Marinkovic et al. (2019) reported that Delta1 secreted from satellite cells and myofibers activates the Notch pathway, inhibiting FAPs adipogenic differentiation. Interestingly, FAPs isolated from chronically dystrophic muscle are unresponsive to Notch-mediated adipogenic inhibition, suggesting that the local environment influences this checkpoint on FAPs fate. Signaling through the retinoic acid receptor has also been shown to enhance proliferation of FAPs and suppress adipogenesis and fibrogenesis (Zhao et al., 2020). Blocking this signaling pathway was shown to promote adipogenesis and to suppress apoptotic clearance of FAPs in regenerating muscle. FAPs express a single central cilium that is also implicated in control of differentiation through its connection to the Hedgehog signaling pathway. Ablation of ciliary signaling in FAPs was shown to inhibit adipogenesis by repressing Hedgehog signaling in a glycerol-induced muscle injury model, resulting in enhanced muscle regeneration (Kopinke et al., 2017).

## Fibroadipogenic progenitors – a contributor to myopathies in broilers?

FAPs have clearly been established to play a role in the formation of myopathies in humans. Whether they play a similar role in broiler breast myopathies has not yet been determined, however evidence supporting this relationship is emerging from the literature. Yablonka-Reuveni et al. (1988) first demonstrated the presence of a non-myogenic cell population that exhibits a fibroblast-like morphology and receptor-mediated binding of PDGF in embryonic chicken breast muscle More recently, transcriptomic studies have reported that PDGFRα is expressed at higher levels in breast muscle of broilers affected with myopathies (Papah et al., 2018; Pampouille et al., 2019; Praud et al., 2020). Expression was significantly increased in birds of a fast-growing commercial line that were affected with white striping or wooden breast in comparison to both unaffected fat-growing counterparts and to a slow-growing line (Pampouille et al., 2019). Malila et al. (2022) also demonstrated that expression of PDGFRα increases with age in breast muscle of fast-growing, but not medium-growing chickens. These findings uncouple the contribution of fast growth *per se* from the association with myopathy incidence and from age-dependent changes in market birds. A genome-wide association study also identified PDGFRα as a candidate gene for contribution to the incidence of white striping in broilers (Pampouille et al., 2019). Based on these findings, and on the established role of FAPs in human myopathies, our lab has begun to characterize this cell population in broilers. We have successfully isolated a PDGFRα- + cell population from pectoralis major that is adipogenic but non-myogenic, and that exhibits morphological features consistent with FAPs from humans and mice (unpublished data). Taken together, these findings suggest that, as in human myopathies, FAPs may contribute to the pathology of breast muscle lesions in broilers.

## Potential implications for alleviating the incidence of breast myopathies in broilers

Because of the clinical relevance, a growing list of pharmacological compounds has been shown to influence the fibrogenic and adipogenic behavior of FAPs in humans (Contreras et al., 2021). Although these types of interventions are not feasible in broilers, they do provide direct evidence that manipulating the cell fate of FAPs reduces or prevents fibrous and fatty infiltration that characterize myopathic lesions. Accordingly, this population may hold new approaches to reduce the development of myopathies in broilers. Dietary means to manipulate FAPs and alleviate myopathies may be feasible based on previous studies in humans and mice. In dystrophies such as DMD, FAPs undergo mitochondrial alterations that compromise oxidative metabolism and a shift from reliance on fatty acid oxidation towards use of glycolysis and the pentose phosphate pathway to derive cellular energy (Vila et al., 2017). Interestingly, this Warburg-like resembles the metabolic consequences of hypoxia that have been linked to broiler breast muscle myopathies. This detrimental metabolic rewiring of FAPs promotes FAPs proliferation and adipogenesis. Restoring fatty acid oxidation by treatment with metformin, which induces fatty acid oxidation by activating AMPK, or by a short-term high fat diet corrects the metabolic and phenotypic defect (Pala et al., 2018; Reggio et al., 2020). Suppression of AMPK signaling through genetic means has also been shown to induce fibrogenic differentiation of FAPs (Liu et al., 2023). Dietary or production strategies that promote fatty acid oxidation or AMPK activation may therefore be a means to influence the behavior of FAPs in broilers. Numerous plant-based sources of polyphenols have been shown to activate AMPK (Ferreira et al., 2024), including in broilers (Huang et al., 2017; Wan et al., 2021; Ding et al., 2023), although their specific effects on fatty and fibrotic infiltrates of breast muscle are unknown. Nitric oxide has also been shown to inhibit FAPs adipogenesis and intramuscular fat accumulation in rodent models (Cordani et al., 2014). *In vivo* treatment with molsidomine, a NO donor, reduced fibrosis and adipogenesis, and lesions in mdx mice, a model of DMD (Cordani et al., 2014). Conversely, blocking NO synthesis with a nitric oxide synthase inhibitor, L-NAME, increased fibrotic tissue deposition and reduced satellite cell activation during muscle regeneration (Filippin et al., 2011). Interestingly, increased activity of the arginine-citrulline-NO synthesis pathway has been associated with reduced severity of wooden breast in broilers, although this was not specifically associated with effects on FAPs (Greene et al., 2019). In addition, including a stabilized form of arginine (inositol-stabilized arginine silicate) that preferentially increases NO production in broiler diets was recently shown to reduce the incidence of wooden breast (Meyer and Bobeck, 2023).

## Summary

FAPs have now been established as the cellular source of fibrofatty infiltrates in human muscular dystrophies. Moreover, multiple studies have demonstrated that the fate of FAPs is a promising therapeutic target for reducing the incidence and progression of these disorders. The extensive histological similarities between myopathic lesions in humans and those found in broiler breast muscle myopathies strongly suggests that this cell type plays a similar role in development of wooden breast, white striping, and other challenges to broiler production. Accumulating evidence from gene expression studies supports this causative role, which suggests new means to potentially alleviate the incidence of myopathies in broilers. Collectively, these findings warrant characterization of this novel cell type as a potential new target of production and dietary approaches to reduce the incidence of breast muscle lesions.
